# Estimated cost of comprehensive syringe service program in the United States

**DOI:** 10.1371/journal.pone.0216205

**Published:** 2019-04-26

**Authors:** Eyasu H. Teshale, Alice Asher, Maria V. Aslam, Ryan Augustine, Eliana Duncan, Alyson Rose-Wood, John Ward, Jonathan Mermin, Kwame Owusu-Edusei, Patricia M. Dietz

**Affiliations:** 1 Division of Viral Hepatitis, National Center for HIV, Viral Hepatitis, STD and TB Prevention, CDC, Atlanta, GA, United States of America; 2 Office of the Director, National Center for HIV, Viral Hepatitis, STD and TB Prevention, CDC, Atlanta, GA, United States of America; Medizinische Universitat Wien, AUSTRIA

## Abstract

**Objective:**

To estimate the cost of establishing and operating a comprehensive syringe service program (SSP) free to clients in the United States.

**Methods:**

We identified the major cost components of a comprehensive SSP: (one-time start-up cost, and annual costs associated with personnel, operations, and prevention/medical services) and estimated the anticipated total costs (2016 US dollars) based on program size (number of clients served each year) and geographic location of the service (rural, suburban, and urban).

**Results:**

The estimated costs ranged from $0.4 million for a small rural SSP (serving 250 clients) to $1.9 million for a large urban SSP (serving 2,500 clients), of which 1.6% and 0.8% is the start-up cost of a small rural and large urban SSP, respectively. Cost per syringe distributed varied from $3 (small urban SSP) to $1 (large rural SSP), and cost per client per year varied from $2000 (small urban SSP) to $700 (large rural SSP).

**Conclusions:**

Estimates of the cost of SSPs in the United States vary by number of clients served and geographic location of service. Accurate costing can be useful for planning programs, developing policy, allocating funds for establishing and supporting SSPs, and providing data for economic evaluation of SSPs.

## Introduction

Syringe services programs (SSPs) are community prevention programs that provide access to sterile needles and syringes at no cost to their clients and facilitate safe disposal of used needles and syringes [1–3]. SSPs also offer a range of other prevention services, such as education on safe injection practices, wound care, overdose prevention with naloxone, referral to substance use disorder treatment, and testing for infections like human immunodeficiency virus (HIV), hepatitis C virus (HCV), and hepatitis B virus (HBV) [2)]. In addition, these programs provide linkage to medical services, such as HIV or HCV treatment, referral to mental health services, and onsite or referral to hepatitis A and B vaccination. SSPs, especially when combined with medication-assisted treatment for opioid use disorder, can play a substantial public health role by preventing acquisition and transmission of blood borne infections, including HIV and HCV [4–7]. Use of SSPs has also been associated with reduced frequency of injection [8]. SSPs are beneficial to the community by reducing used syringes in public spaces, which carry the risk of accidental needle prick injury [9]. SSPs do not increase crime or drug use [10].

The recent increase in injection drug use and HCV infections related to the national epidemic of non-prescription use of opioids has increased the need for SSPs [11–15]. As of 2016, there were 228 SSPs registered in the United States, located primarily in urban areas, the West Coast, and the Northeast [16]. However, the current number of SSPs is not adequate to address the rising number of persons who inject drugs (PWIDs). The majority of US counties identified as vulnerable for rapid transmission of HIV and/or HCV are concentrated in rural areas where few SSPs exist [17]. In fact, only 20% of young persons who received a diagnosis of HCV infection, for whom injection drug use is a major risk factor, lived within 10 miles of an SSP [17].

Funding for SSPs currently operational in the United States is limited, and therefore the cost of establishing and operating an SSP that provides comprehensive service is not well known [15]. Detailed knowledge of cost is important for planning, assessing allocation of funding and grant provision towards setting up services in affected communities, and providing data for conducting economic evaluation. Obtaining estimates from surveys would be time-consuming and very costly. Additionally, given that most SSPs operate with free and/or under-priced and inconsistent inputs, estimates from surveys might not depict the actual costs involved. In this study, we sought to determine the cost of a comprehensive SSP in urban, suburban, and rural locations in the United States from the service payer perspective using a bottom-up budgeting approach––identifying individual service/input cost, categorizing and aggregating them.

## Methods

For the purpose of this study, we defined a ‘comprehensive SSP’ as a program that provides sterile injection equipment and disposal of used equipment, and offers a variety of preventive and medical services including: onsite HIV and HCV counseling and testing; first aid/wound care; overdose prevention education and naloxone kits; onsite provision or referral to hepatitis A and hepatitis B vaccination; referrals to sexually transmitted infection (STI) testing, tuberculosis (TB) screening, substance use disorder treatment, and care and treatment of HBV, HCV, and HIV. Comprehensive SSPs may also provide referrals to primary care, mental health services, reproductive health services, and social services.

To determine the cost of a comprehensive SSP to the service provider, we first categorized cost into the following groups: one-time facility and equipment, personnel, operational, prevention services, and onsite medical/testing services. Our cost estimate focused on the first year cost referred to as one-time facility and equipment cost, which is not recurrent and other costs which will be incurred in the first year and thereafter. These costs do not include societal costs, which includes patient travel costs and volunteer time costs. One-time costs were assumed to be incurred in the first year only, while the other categories of cost were assumed to be incurred annually. Because the total costs of comprehensive SSPs will vary by the number of clients served per year and by their geographic location, we estimated total costs for three client-volume categories and three geographic areas. We defined three client volume-based categories as small (250/year), medium (1,250/year), and large (2,500/year). We defined geographic areas using census region-based classifications for estimation of cost of services and staff salaries as urban (> 1.0 million), suburban (50,000–1.0 million) and rural (<50,000) [18]. We used a three-step approach (described below) to obtain estimates that were representative of the three geographic areas.

We obtained budgets from SSPs in Maine, California, Kentucky and Atlanta, and spoke with SSP directors from these programs to ensure that our assumptions of services provided matched what programs offered, and to familiarize ourselves with current SSP budgets. The interviews with directors confirmed that SSPs are under-resourced, and that employee salaries generally were not in line with other non-profit agencies.

### One-time facility and equipment costs

The one-time costs include lease/rent deposit (i.e., cost of the first or last month rent), office furniture, and office equipment (e.g., computers, phones, modems). We assumed each SSP needs a fax machine (including a copier, a scanner, and a printer), a modem, a router, two phones, and a mobile phone. The need in furniture items and computers is determined by SSP size, whereas the lease/rent deposit is determined by SSP size and location (S1 Appendix). The cost of such items was determined from online sources that ship their products anywhere in the United States, and either offer wholesale discount or match prices of their competitors to ensure competitive prices. This cost will not be incurred in subsequent years by the program.

### Personnel costs

Personnel costs include wage/salaries of an SSP staff, employee benefits/insurance, staff training and education, and volunteer incentives (S2 Appendix). We assumed each SSP needs a full-time director, a part-time street outreach specialist, and a part-time peer navigator. The need in other personnel (i.e., part-time counselors, a part-time nurse, or a part-time accountant) is determined by SSP size. Detailed job description, staffing needs, and wage estimates are provided in S2 Appendix. Employee benefits/insurance accounts for 30% of personnel’s total compensation (S2 Appendix). Per communications with SSPs staff, we assumed the cost of staff training and education ranges from $2,000 to $3,000 and volunteers’ incentives range from $600-$1200 US dollars per year (S2 Appendix).

We used a three-step approach to obtain wage estimates that were representative of the three geographic areas (urban, suburban, and rural). First, we identified census regions with the highest and lowest overall prices for goods and services—the Northeast and Midwest, respectively [18]. Second, we focused on the wage data for Community and Social Services (CSS) employees who were the primary occupational category for SSPs. Within selected census regions, we identified states that reported the lowest and the highest CSS wages—Kansas (Midwest) and New Jersey/New York (Northeast), respectively [19, 20]. Finally, in those states we identified wages for CSS employees in specific geographic areas (urban, suburban, and rural) [18]. The lower bound wage estimates for each geographic area were obtained from Kansas state—Kansas City (urban), St. Joseph/Wichita (suburban), Southwest/Southeast Kansas (rural). The upper bound wage estimates were obtained from New York state—New York City (urban), Rochester (suburban), and Capital/Northern New York (rural). We used the midpoints of the lower and upper bounds as our final estimates.

### Operational costs

These costs are associated with lease/rent, insurance, utilities, mail services, and janitorial services. We used the three-step approach described above to determine office rent in urban, suburban and rural areas, where the upper and lower bounds of the estimates represent geographic areas with the highest and lowest cost of goods/services and property rent, respectively. In urban areas, we excluded high-end corporate properties (e.g., offices located in iconic skyscrapers or office parks). Instead, we considered offices that provided storage space. For each location, we obtained estimates for three size options that approximate SSP size: 1–4 people (75–300 square feet; may accommodate a small SSP), 5–9 people (375–675 square feet; medium SSP), and 10–24 people (740–1800 square feet; large SSP). The annual cost of renting an unfurnished office was determined from online sources that reported rental prices across the country and allowed potential renters to select a specific location (S3 Appendix). The costs of internet, phone plans, and web hosting were determined from online sources using the methodology described for estimating one-time costs (S3 Appendix).

### Prevention services costs

These costs are associated with sterile syringes and needles and other injecting equipment such as cotton filters, sterile water, and cookers, as well as naloxone, hazardous waste management, and sharps containers. The total cost of injection equipment per person per year was calculated based on the estimated average number of syringes to be distributed to each client per year. For our analysis, we used an average of 600 (range 400–800) syringes per person per year, assuming each client injects multiple times a day, and may take an ‘injection break’ during a twelve month period for personal (i.e. drug treatment) or legal (i.e. incarceration) reasons. Our choice of 600 syringes was between the World Health Organization recommendation of 300 syringes per person per year and a published estimate of 720–900 needles per person per year [21–23]. The costs of hazardous waste management and sharps containers were based on the overall estimated number of syringes used and returned to the SSP. This cost item also includes provision of overdose prevention kits and two units of naloxone to all participants of the program as allowed by local laws. Detailed description of the cost components is provided in S4 Appendix.

### Onsite medical and testing services cost

Supplies needed for HIV and HCV point-of-care testing and hepatitis A and B vaccinations were determined based on testing recommendations and SSP size. Detailed description of the cost components is provided in S5 Appendix. Where testing is recommended to all, we estimated cost assuming 100% coverage. However, if recommendations varied based on previous exposure or test result, we estimated the proportion for whom the service was recommended (e.g., hepatitis B vaccination is recommended for unvaccinated individuals only) [24]. Cost of pregnancy test was estimated assuming one test per person per year where 50% of the served population will be female.

### Mobile units

Besides providing services at fixed sites, an SSP can elect to have an additional mobile unit depending on available resources, location, local epidemiology of drug use, and catchment area for the service. We estimated the one-time cost of a mobile SSP and assumed that personnel from the fixed program will operate the mobile unit. Operational costs of a mobile unit (vehicle registration, insurance, furniture, maintenance, gas, and storage space) were estimated in the same way as other operational costs (see above). Detailed description of the cost components for a mobile van is provided in S6 Appendix.

Based on the overall total costs and number of clients, we calculated the cost per client per year, as well as the proportional contribution of costs by category to the total cost. Detailed information on the methods and sources of the data are presented in S1–S5 Appendices. All costs were in 2016 US dollars.

## Results

The total cost for the first year of a comprehensive SSP ranged from $0.4 (range $0.3-$0.6) million for a small rural program to $1.9 (range $1.4-$2.3) million for a large urban SSP (Table 1). The components of a comprehensive SSP, detailed information on cost by item, and sources of costs are provided in Appendix A. The estimated cost of SSPs per syringe distributed varied from approximately $1 for a large rural SSP to $3.4 for a small urban SSP, and the estimated cost per client per year varied from $661 for a large rural SSP to $2008 for a small urban SSP. Based on volume of clients, the estimated number of syringes to be distributed by small, medium, and large SSPs were 0.15 million, 0.75 million, and 1.5 million per year, respectively (Table 2). The cost per syringe distributed and per client per year was almost three times as high for small SSPs compared to large SSPs. The percent increase in cost per syringe distributed from a large urban to a small urban SSP reached 183%.

**Table 1:** Estimated costs of a comprehensive syringe service program (SSP) by size and geographic location, United States (in $1,000 2016 US dollars).

	Large*SSPs cost midpoint**(range)***			Medium* SSPs cost midpoint (range)			Small* SSPs cost midpoint (range)		
Category	Rural	Suburban	Urban	Rural	Suburban	Urban	Rural	Suburban	Urban
**Total Cost, including**	**1698.7 (1234.2–2163.1)**	**1732.9 (1275.9–2189.8)**	**1855.0 (1363.2–2346.8)**	**986.3 (722.1–1250.5)**	**1012.8 (757.5–1268.0)**	**1102.5 (825.4–1379.7)**	**449.2 (342.1–556.2)**	**470.6 (370.2–571.0)**	**546.8 (428.7–664.8)**
One-time cost^1^ (Start-up only)	13.2 (10.3–16.1)	13.6 (10.6–16.5)	15.4 (10.7–20.2)	9.6 (7.1–12.2)	9.8 (7.3–12.2)	10.5 (7.3–13.6)	7.3 (5.7–9.0)	7.4 (5.7–9.1)	7.7 (5.6–9.8)
Personnel^2^	376.3 (315.0–437.7)	408.3 (356.6–459.9)	504.2 (439.1–569.2)	305.0 (254.2–355.7)	329.8 (287.5–372.1)	410.5 (355.1–465.8)	256.8 (215.0–298.6)	278.3 (243.2–313.5)	350.5 (301.6–399.4)
Operational^3^	144.9 (108.8–181.1)	149.4 (112.8–186.0)	171.7 (113.3–230.1)	67 (48.4–85.5)	69.0 (50.6–87.4)	77.3 (50.8–103.9)	27.8 (19.5–36.1)	28.2 (19.5–36.9)	31.9 (19.6–44.3)
Prevention services^4^	1006 (693.0–1319.0)	1003.9 (688.9–1319.0)	1003.9 (688.9–1319.0)	503.0 (346.6–659.5)	503.0 (346.6–659.5)	503 (346.6–659.5)	100.6 (69.3–131.9)	100.6 (69.3–131.9)	100.6 (69.3–131.9)
Onsite medical/testing services^5^	112.9 (82.7–143.0)	112.9 (82.7–143.0)	112.9 (82.7–143.0)	56.4 (41.4–71.5)	56.4 (41.4–71.5)	56.4 (41.4–71.5)	11.3 (8.3–14.3)	11.3 (8.3–14.3)	11.3 (8.3–14.3)
Mobile van unit^6^	45.4 (24.4–66.3)	44.8 (24.3–65.3)	44.8 (24.3–65.3)	45.4 (24.4–66.3)	44.8(24.3–65.3)	44.8(24.3–65.3)	45.4 (24.4–66.3)	44.8 (24.3–65.3)	44.8 (24.3–65.3)

*Large SSPs serve 2,500 clients per year, medium SSPs serve 1,250 clients per year, and small SSPs serve 250 clients per year.

**Midpoint cost refers to average cost of the highest and lowest costs.

***Range refers to the upper and lower bounds of estimated costs of SSPs, and they reflect geographic differences in the cost of goods and services, rent, and wages in the United States. The lower bound estimates were obtained from Kansas state—Kansas City (urban), St. Joseph/Wichita (suburban), and Southwest/Southeast Kansas (rural). The upper bound estimates were obtained from New York state—New York City (urban), Rochester (suburban), and Capital/Northern New York (rural).

^1^ One-time costs include lease/rent deposit, office furniture, and office equipment (e.g., items such as computers, mobile phones, office furniture, and modems).

^2^ Personnel categories include a program director, a part-time accountant, peer navigators, a part-time nurse, and counsellors.

^3^ Operational costs are associated with lease/rent, insurance, utilities, mail services, and janitorial services.

^4^ Prevention services costs are associated with sterile syringes and needles and other injecting equipment such as cotton filters, sterile water, and cookers, as well as naloxone, hazardous waste management, and sharps containers.

^5^ Onsite/medical testing services costs include point of care testing for hepatitis C virus and human immunodeficiency virus, hepatitis A and B vaccination, wound care, and pregnancy tests.

^6^ Mobile van unit costs include the cost of a van, registration, maintenance, gas, storage, and insurance.

^2–5^ Represent annual costs.

**Table 2:** Estimated per syringe and per client costs of a comprehensive syringe service program (SSP) by size and geographic location, United States (in 2016 US dollars).

	Large* SSPs cost			Medium* SSPs cost			Small* SSPs cost		
Category	Rural	Suburban	Urban	Rural	Suburban	Urban	Rural	Suburban	Urban
**Cost, ($)/syringe**	1.1	1.1	1.2	1.3	1.3	1.5	2.7	2.8	3.4
**Cost, ($)/year/** **client**	661.3	675.2	724.1	752.6	774.3	846.2	1,615.1	1,703.0	2,007.7
**Number of syringes distributed/year**	1,500,000			750,000			150,000		

* Large SSPs serve 2,500 clients per year, medium SSPs serve 1,250 clients per year, and small SSPs serve 250 clients per year.

Table 3 demonstrates the contribution of each category of cost to the overall total cost. Approximately half (54.2%-57.9%) of the cost of large SSPs was associated with preventive/medical services, whereas for small SSPs most (57.2%-64.1%) of the cost was driven by personnel. Prevention services (e.g., needles and syringes, sterile water, naloxone, and hazardous waste disposal) comprised as much as 45.6%-57.9% of the costs of a large or medium SSPs, yet, for a small SSP they represented about 20% of the cost. The cost that will not be covered by federal funds (materials used in the preparation and injection of drugs) is approximately 28% for large SSPs and 11% for small SSPs. The cost of HCV testing was about 2.1% of the overall cost of a large SSP (data not shown). The subsequent year costs of SSP will not include the one-time cost, thus is expected to be 0.8–1.6% lower than the first year cost. After the start-up year, the cost of SSP is expected to decrease further as some activities are one time interventions (e.g., hepatitis A and B vaccination), and positive impact of the program could influence frequency of drug use thus decreasing number of injection equipment supplied.

**Table 3:** Proportional contribution (percentages) of each cost category to the overall total cost of a comprehensive syringe service program (SSP) by size and geographic location, United States.

	Large* SSPs			Medium* SSPs			Small* SSPs		
Components of cost	Rural %	Suburban %	Urban %	Rural %	Suburban %	Urban %	Rural %	Suburban %	Urban %
One-time cost^1^ (Start-up only)	0.8	0.8	0.8	1.0	1.0	1.0	1.6	1.6	1.4
Personnel^2^	22.1	23.6	27.2	30.9	32.6	37.2	57.2	59.1	64.1
Operational^3^	8.5	8.6	9.3	6.8	6.8	7.0	6.2	6.0	5.8
Prevention services^4^	59.2	57.9	54.2	51.0	49.7	45.6	22.4	21.4	18.4
Onsite medical/testing services^5^	6.7	6.5	6.1	5.7	5.6	5.1	2.5	2.4	2.1
Mobile van unit^6^	2.7	2.6	2.4	4.6	4.4	4.1	10.1	9.5	8.2
**Total**	**100.0**	**100.0**	**100.0**	**100.0**	**100.0**	**100.0**	**100.0**	**100.0**	**100.0**

* Large SSPs serve 2,500 clients per year, medium SSPs serve 1,250 clients per year, and small SSPs serve 250 clients per year.

^1^ One-time costs include lease/rent deposit, office furniture, and office equipment (e.g., items such as computers, mobile phones, office furniture, and modems).

^2^ Personnel categories include a program director, a part-time accountant, peer navigators, a part-time nurse, and counsellors.

^3^ Operational costs are associated with lease/rent, insurance, utilities, mail services and janitorial services.

^4^ Prevention services costs are associated with sterile syringes/needles and other injecting equipment such as cotton filters, sterile water, and cookers, as well as naloxone, hazardous waste management, and sharps containers.

^5^ Onsite/medical testing services costs include point of care testing for hepatitis C virus and human immunodeficiency virus, hepatitis A and B vaccination, wound care, and pregnancy tests.

^6^ Mobile van unit costs include the cost of a van, registration, maintenance, gas, storage, and insurance.

^2–5^ Represent annual costs.

## Discussion

We estimated the cost of establishing and maintaining a comprehensive SSP for different geographic locations in the United States. With an average cost of $1 to $3 per syringe, a syringe service program can inexpensively prevent serious infectious diseases in those who are not infected, help identify and link those already infected to care, and prevent overdose and other related harms associated with the opioid epidemic by linking to preventive services (such as referral to treatment for opioid use disorder). The cost of a comprehensive SSP depends on the program size, location, and types of services provided. Some services are intrinsically costly (e.g., provision of naloxone), whereas other services require delivery by specially trained staff (e.g., peer navigation, conducting HIV and HCV tests, and providing wound care). Larger SSPs serve more clients and thus, incur higher total costs. However, costs per-syringe distributed and per-client were considerably lower for large SSPs compared to small SSPs.

Our cost estimate was comparable to prior studies in the United States and Mexico. The cost per syringe distributed was $0.97 in the US whereas the cost in Mexico was $0.76 [25, 26]. Based on our estimates, at a cost of approximately $724 per client per year, a large, urban, comprehensive SSP can provide services that prevent transmission of blood borne infections, counselling for substance use disorder, and referral for treatment for HIV and HCV infections. The cost of an SSP that reduces HIV or HCV transmission via injecting drug use could be as small as $2 ($724/365) per person per day. Furthermore, with this cost, SSPs can connect PWID to treatment programs for substance use disorders, prevent overdose by providing overdose prevention education and supply of naloxone.

The federal appropriation act of 2016 allowed for the use of federal funds to establish or expand existing SSP services [24]. However, federal funds cannot be expended for materials used for the preparation and injection of drugs [27]. In our estimate the cost that will not be covered by federal funds amounts to approximately 28% for large SSPs and 11% for small SSPs.

A major component of the cost of SSPs is salaries and benefits. For many existing SSPs, volunteers, including former and recovering PWID, provide some of the staffing. Thus, our estimates of salaries and benefits will be higher than SSPs that rely on volunteers for key staff positions. Personnel costs will depend on what positions volunteers fill, as well as costs related to volunteer incentives, which was not considered in our analysis. Larger SSPs will need more paid staff than smaller SSPs. Overall, the number and type of expertise of staff will have a significant impact on the cost of a program. Our cost estimate assumes the SSP will provide a minimum of 40 hours of service at the fixed location.

Recent studies demonstrate that less than 10% of young persons with recent HCV diagnoses reside within 10 miles of an SSP [17], and those who do have access to an SSP suffer from stigma, fear of identification, and concern over confidentiality. These barriers may be overcome with wider use of alternative services including mobile SSP units, especially in rural settings where the number of PWIDs has been on the rise [13].

This study has some important limitations. The program size and the amount of injection equipment per person are based on estimates. In addition, the number of injections per person may vary by the type of drug commonly used by the local PWID population. However, most PWIDs who attend SSPs are injecting heroin and our estimates are based on this premise. Additionally, we may have overestimated the cost of programs for a variety of reasons. First, we assumed full staffing. Second, we did not account for secondary exchange of syringes (distribution of injection equipment by peers) that would likely reduce staffing needs. Third, we assumed full coverage for some services (such as testing and vaccination) for eligible clients. We did not include depreciation cost. However, given that the depreciation-eligible items make up less than 10% of the total cost, this omission would not have substantial impact on the first-year estimate.

The cost of SSPs in urban, suburban, and rural areas varied by size and geography. These findings can inform implementers and funders, and can be used as a benchmark for future estimates, as well as by policy makers when deciding to start and operate an SSP. This information can also contribute to further economic evaluation studies of this effective public health prevention tool. SSPs will provide an access point to target the population disproportionately affected by the opioid epidemic and the related morbidity and mortality and may reduce the societal cost of opioid use disorder.

## Supporting information

S1 Appendix One-time cost methods and sources. (DOCX)

S2 Appendix Personnel cost methods and sources. (DOCX)

S3 Appendix Operational cost methods and sources. (DOCX)

S4 Appendix Prevention/medical services cost methods and sources. (DOCX)

S5 Appendix Onsite medical/testing services cost methods and sources. (DOCX)

S6 Appendix Mobile van cost methods and sources. (DOCX)
