# Supplementary material for: Estimated cost of comprehensive syringe service program in the United States
Source: PLoS One. 2019 Apr 26;14(4):e0216205. doi: 10.1371/journal.pone.0216205 (PMC6485753; doi:10.1371/journal.pone.0216205)
Supplement: S1 Appendix — (DOCX) [file pone.0216205.s001.docx]

**S1 Appendix. One-time cost methods and sources**

| **One-time Costs (Start up only)** | **Quantity and Justification** | **Estimated Unit Cost** | **Source** |
| --- | --- | --- | --- |
| Computers | Assumes 4 computers for a large SSP, 3 for meduim SSP, and 1 for small SSP. Computers used for communication, data collection, and administrative takes. | $500-$1200 | Amazon (search term "computer"; <https://www.amazon.com/s/ref=nb_sb_noss_2?url=search-alias%3Daps&field-keywords=computer&rh=i%3Aaps%2Ck%3Acomputer>) and Google (search term "average cost of computer"; <http://gizmodo.com/5033865/study-average-mac-computer-price-more-that-twice-that-of-average-pc.>)Accessed 19July2017 |
| Phone | Estimating 2 phones per SSP. Phones used for communication with clients, employees, and other stake holders. | $50-$200 | Amazon (search term: "Office Phones"); Personal Communication with SSPs. Link: <https://www.amazon.com/s/ref=nb_sb_noss_1?url=search-alias%3Daps&field-keywords=office+phone&rh=i%3Aaps%2Ck%3Aoffice+phone> ); Personal Communication with SSPs. Accessed 19July2017 |
| Fax machine, including Copier, Scanner, and Printer | Assumes 1 fax machine per SSP. Used for copying, faxing, printing and scanning forms. | $130-$2000 | Amazon (search term: "fax machine"; <https://www.amazon.com/s/ref=nb_sb_noss_1?url=search-alias%3Daps&field-keywords=fax+machine&rh=i%3Aaps%2Ck%3Afax+machine>), Google (average cost of fax machine; <http://smallbusiness.costhelper.com/fax-machine.html>); Personal Communication with SSPs. Link: and accessed 19Jul2017 |
| Modem/router | Assumes 1 modem and 1 router per SSP. Necessary for program Wifi accessibility. | $120-$220 | Amazon (search term: "modem and router"; [https://www.amazon.com/s/ref=nb_sb_ss_c_1_13?url=search-alias%3Daps&field-keyword s=modem+and+router&sprefix=modem+and+rou%2Caps%2C139&crid=47L5L7NNR0WX](https://www.amazon.com/s/ref=nb_sb_ss_c_1_13?url=search-alias%3Daps&field-keyword%20s=modem+and+router&sprefix=modem+and+rou%2Caps%2C139&crid=47L5L7NNR0WX)) Accessed 19Jul2017 |
| Mobile phone | Assumes 1 phone per SSP. For peer navigators and outreach activity to communicate with clients. | $100-$300 | Amazon (search term: "mobile phone"; <https://www.amazon.com/s/ref=nb_sb_noss_1?url=search-alias%3Daps&field-keywords=mobile+phone>) Accessed Date: 19Jul2017 |
| Lease/rent deposit | First month/Last month, depending on lease agreement. | Cost of first month/last month rent | See Appendix S1C. |
| Office furniture | Funds used to furnish program office. Does not include mobile van furniture. Assumes the following items for small (S), meidum (M), and large (L) SSPs:  *Waiting Room Space:  Waiting room chairs:  5 chairs (S), 10 (M), 15(L)  Table for educational materials: 1 table (S), 1 (M), 2(L)  Table for food/coffee:  1 table (S), 1 (M), 1(L)  Table for client intake:  1 table (S), 1 (M), 2(L)  Cabinet for syringes/harm reduction supplies:  1 cabinet (S), 1 (M), 2(L)  * Counselling and testing room:  Testing tables (1/counsellor):  1 table (S), 1 (M), 2(L)  Chairs (2/table for counsellor + client):  2 chairs (S), 2 (M), 4(L)  Cabinet for test kits and basic medical supplies:  1 cabinet (S), 1 (M), 2(L)  *Medical care/nurse room:  Phlebotomy chair:  1 chair (S), 1 (M), 1(L)  Cabinet for medical supplies:  1 cabinet (S), 1 (M), 1(L)  Table for processing:  1 table (S), 1 (M), 1(L)  Chairs (at least 1 for Nurse):  2 chairs (S), 2(M), 2(L)  Small refigerator (for vaccines):  1 refigerator (S), 1 (M), 1(L)  Exam table:  0 tables (S), 0 (M), 1(L)  *Administrative space:  Desks with file cabinets:  3 desks (S), 4(M), 5(L)  Chairs (at least 1/desk):  3 chairs (S), 4(M), 5(L)  Cabinet for outreach/educational materials:  1 cabinet (S), 1 (M), 1(L) | $39 ^ƚ^  $116 ^ƚ^  $116 ^ƚ^  $116 ^ƚ^  $948 ^ƚ ƚ^  $88 ^ƚ^  $39 ^ƚ^  $462 ^ƚ ƚ^  $289 ^ƚ ƚ^  $462 ^ƚ ƚ^  $88 ^ƚ^  $129 ^ƚ^  $175 ^ƚ^  $88 ^ƚ^  $216 ^ƚ^  $129 ^ƚ^  $172 ^ƚ^ | ^ƚ^ Wayfair website (is an online website and ships everything for free): <https://www.wayfair.com/>  ^ƚ^ ^ƚ^ Tigermedical website (matches prices of its competitors and ships most items for free): <http://www.tigermedical.com/>.  Access Date: 19Jul2017 |

* Personal Communication with SSP – We obtained the budgets of 7 SSPs located in rural, suburban and urban locations in California, Maine, Atlanta, and Kentucky, and interviewed the SSP directors to understand programs needs and budgetary concerns.
