# Supplementary material for: Estimated cost of comprehensive syringe service program in the United States
Source: PLoS One. 2019 Apr 26;14(4):e0216205. doi: 10.1371/journal.pone.0216205 (PMC6485753; doi:10.1371/journal.pone.0216205)
Supplement: S2 Appendix — (DOCX) [file pone.0216205.s002.docx]

**S2 Appendix. Personnel cost methods and sources**

| **Personnel Costs** | **Quantity and Justification** | **Estimated Unit Cost** | **Source** |
| --- | --- | --- | --- |
| Project (executive) director | Full time equivalent (FTE), 40 hours per week. Duties including overseeing the SSP through planning, hiring, fundraising, and marketing. Detailed job description and wage estimates ─ see "Medical and Health Services Managers" (occupational code 11-9111) in Bureau of Labor Statisitcs. | Estimated for 40 hours per week.  Urban SSP: $123,305 ($101,120-$145,590);  Suburban SSP: $91,205 ($79,040-$103,370);  Rural SSP: $85,600 ($70,230-$100,97) | Bureau of Labor Statistics. Occupational Employment and Wages, May 2016: Medical and Health Services Managers <https://www.bls.gov/oes/2016/may/oes119111.htm> |
| Street Outreach Specialist/Peer Navigator | FTE, 40 hours per week total (20 hours per week Outreach Specialist; 20 hours Peer Navigator). Street outreach specialist will usually have a bachelors degree in health promotion from a University. Peer navigator will provide assistance and support for clients to navigate through substance use related challenges, as well as making informed healthcare decisions; recruited locally and likely is an ex-PWID with ties in the community. Detailed job description and wage estimates ─ see "Community Health Workers" (occupational code 21-1094) in Bureau of Labor Statisitcs. | Estimated for 40 hours per week.  Urban SSP: $40,060 ($35,950-$44,170);  Suburban SSP: $34,510 ($29,080-$39,940);  Rural SSP: $32,095 ($25,330-$38,860) | Bureau of Labor Statistics. Occupational Employment and Wages, May 2016: Community Health Workers <https://www.bls.gov/oes/current/oes211094.htm> |
| Counselors | Large SSP: 60 hours per week total (3 counselors each working 20 hours per week). Medium/small SSP: 40 hours per week total (2 counselors each working 20 hours per week). Counselors provide testing and counseling service for HIV, HCV, HBV, STI and TB; provide harm reduction education and referral services to clients. Detailed job description and wage estimates ─ see "Mental Health Counselor" (occupational code 21-1014) in Bureau of Labor Statisiticss. | Estimated for 60 hours per week (large SSP) or 40 hours per week (medium/small SSP).  Urban large SSP: $70,470 ($63,150-$77,790);  Urban medium/small SSP: $46,980 ($42,100-$51,860);  Suburban large SSP: $57,255 ($52,365-$62,145);  Suburban medium/small SSP: $38,170 ($34,910-$41,430);  Rural large SSP: $49,298 ($45,540-$53,055)  Rural medium/small SSP: $32,865 ($30,360-$35,370) | Bureau of Labor Statistics. Occupational Employment and Wages, May 2016: Mental Health Counselors <https://www.bls.gov/oes/2016/may/oes211014.htm> |
| Nurse | Large SSP-20 hours per week, Medium SSP - 15 hours per week, Small SSP - 10 hours per week. Will provide medical services including treatment of abscess and vaccination if available on site; will conduct HIV and HCV rapid testing and blood draw. Detailed job description and wage estimates ─ see "Licensed Practical and Licensed Vocational Nurses" (occupational code 29-2061) in Bureau of Labor Statisitcs. | Estimated for 20/15/10 hours per week for large/medium/small SSP, respectively.  Urban large SSP: $23,820 ($20.340-$27,300);  Urban medium SSP: $17,865 ($15,255-$20,475);  Urban small SSP: $11,910 ($10,170-$13,650);  Suburban large SSP: $19,680 ($19,335-$20,025);  Suburban medium SSP: $14,760 ($14,501-$15,019);  Suburban small SSP: $9,840 ($9,668-$10,013)  Rural large SSP: $19,173 ($18,840-$19,505);  Rural medium SSP: $14,379 ($14,130-$14,629);  Rural small SSP: $9,586 ($9,420-$9,753); | Bureau of Labor Statistics. Occupational Employment and Wages, May 2016: Licensed Practical and Licensed Vocational Nurses <https://www.bls.gov/oes/2016/may/oes292061.htm> |
| Staff accountant | Large SSP - 3 hours per week, Medium/Small SSP - 2 hours per week. The staff accoutant is a contractor that will assist in payroll and tax prep. Detailed job description and wage estimates ─ see "Accountants and Auditors" (occupational code 13-2011) in Bureau of Labor Statisitcs. | Estimated for 3 hours per week (large SSP) or 2 hours per week (medium/small SSP).  Urban large SSP: $7,210 ($7,177-$7,246);  Urban medium/small SSP: $4,807 ($4,785-$4,829);  Suburban large SSP: $5,089 ($4,907-$5,271);  Suburban medium/small SSP: $3,393 ($3,272-$3,514);  Rural large SSP: $4,249 ($4,036-$4,462)  Rural medium/small SSP: $2,833 ($2,691-$2,975) | Bureau of Labor Statistics. Occupational Employment and Wages, May 2016: Accountants and Auditors <https://www.bls.gov/oes/2016/may/oes132011.htm> |
| Volunteers | We did not include the opportunity cost of volunteers’ time. | N/A | N/A |
| Volunteer incentives | Funds used to purchase incentives for volunteers as needed (food, shirts, etc.). Shows appreciation to volunteers for all of their work. | $600-$1200 per year | Estimation |
| Employee benefits/insurance | 30% of total compensation, including healthcare for employees. | 30% of the cost of total compensation. | US Department of Labor (Google Search: average cost of employee benefits and insurance) Link: <https://www.bls.gov/news.release/pdf/ecec.pdf> Accessed date: 19Jul2017 |
| Staff training and education | Staff will be trained upon hire and receive regular update trainings at least once a year. Training include basic training topics including standard operating procedures, referral to medical, substance abuse treatment, mental health, other service agencies, cultural sensitivity, overview of neighborhood concerns, outreach strategies, training secondary exchangers, HIV and viral hepatitis transmission and prevention, overdose prevention, syringe safety/disposal, plan for accidental needlesticks, legal and law enforcement climate. Advanced Training Topics including polysubstance use, conflict resolution and de-escalation, specialized interviewing techniques (e.g., motivational interviewing), principles of case management, abscess and cellulitis treatment and prevention, domestic violence issues, co-occurring mental health and substance use disorders. | $2,000-$3,000 | * Personal Communication with SSPs |
