# Supplementary material for: Estimated cost of comprehensive syringe service program in the United States
Source: PLoS One. 2019 Apr 26;14(4):e0216205. doi: 10.1371/journal.pone.0216205 (PMC6485753; doi:10.1371/journal.pone.0216205)
Supplement: S3 Appendix — (DOCX) [file pone.0216205.s003.docx]

**S3 Appendix. Operational cost methods and sources**

| **Operational Costs** | **Quantity and Justification** | **Estimated Unit Cost** | **Source** |
| --- | --- | --- | --- |
| Rent | For each location used to obtain wage estimates, we estimated office rent in urban, suburban and rural areas. The upper bound of our estimates represented geographic areas with the highest cost of goods/services and property rent (New York state— New York City [urban], Rochester [suburban], and Capital/Northern New York [rural]). By contrast, the lowest bound represented areas with the lowest cost of goods/services and property rent (Kansas state — Kansas City [urban], St. Joseph/Wichita [suburban], and Southwest/Southeast Kansas [rural]). For urban areas, we excluded high-end corporate properties (e.g., offices located in iconic skyscrapers or office parks) and instead focused on ones with storage space. For each locaton, we obtained rent estimates for three size options: 1-4 people (75-300 square feet; sufficient to accommodate a small SSP), 5-9 people (375-675 square feet; sufficient to accommodate a medium SSP), and 10-24 people (740-1800 square feet; sufficient to accommodate a large SSP). We reviewed several websites that report rental prices from all over the country and permit picking a specific location. From those websites, we selected Loopnet.com as an example. | Estimated per office per year.  Urban large SSP: $32,175 ($7,650-$56,700);  Urban medium SSP: $12,066 ($2,869-$21,263);  Urban small SSP: $5,363 ($1,275-$9,450);  Suburban large SSP: $9,864 ($7,128-$12,600);  Suburban medium SSP: $3,699 ($2,673-$4,725);  Suburban small SSP: $1,644 ($1,188-$2,100);  Rural large SSP: $5,400 ($3,150-$7,650)  Rural medium SSP: $2,025 ($1,181-$2,869)  Rural small SSP: $900 ($525-$1,275) | <http://www.loopnet.com> Assessed Date: 19Jul2017 |
| Insurance | This includes general liability – (Workers Compensation, Auto insurance for van, Property, excess). Ranges are estimated. | Calculated by proporation of SSP Size, comparing large/small to medium. Original estimates 5000-7000. Small SSP ($1000-$1400) Medium SSP ($5000-$7000), Large SSP ($10000-$14000) | * Personal Communication with SSPs and Estimination. |
| Utilities | Estimating utilities to cost $800-$1200 per month. This will include water and power (electricty or gas). | $800-$1200 per month. This will be the value for the Small SSP utilties costs, and will be used as references for medium/large costs. Proportion will be calculated by square footage size, which can be find on the rent tab of "Wages Salaries Rent" Excel Document. The upper estimates of square footage for each size will be used to calculate proproations. Costs per Month: Small SSP ($800-$1200), Medium SSP ($1800-2700), Lage SSP ($4800-7200). | Estimation |
| Tax preparation and audit costs | Funds used to prepare for audits and taxes. | $250-$6000 | * Personal Communication with SSPs |
| Bank administrative fee (yearly) | Funds used to pay bank fees. Price ranges are estimates. | $0.00-$400 | * Personal Communication with SSPs |
| Office supplies | Funds used for office supplies (paper, pens, etc.) for program. | Monthly cost range from $19-$167. Costs will be proportional to the size of the SSP comparing large/medium to small. Small SSP ($19-167), Medium SSP ($95-835), Large SSP (190-1670) | Financial website Bundle; Personal Communication with SSPs. Link: <http://www.newtekone.com/2011/08/18/how-to-get-office-supplies-on-the-cheap/> Accesed Date: 19Jul2017 |
| Internet and phone plans | Monthly cost for internet and phone bundle. | Ranges from $90-$260. Different providers should offer compatible speeds. Pricing depends on speed of interenet. | Comcast Business Website. Link: <https://business.comcast.com/> Access Date: 19Jul2017 |
| Food (for clients) | Food and drinks to provide to clients as they come to receive SSP service. | Amount of food proportional to number of clients. Small SSP Food = $90-$140. Medium SSP Food = $450-$700. Large SSP Food = $900-$1400. | Estimation |
| Household (cleaning supplies) | Funds used to purchase supplies to maintain and clean program office space. | $200-400/year. Proportion based on SSP sq ft size using the large sqaure foot amount for each SSP. Small (300 sq ft), medium (675 sq ft), large (1800). Small range ($200-$400), Medium range ($450-$900), Large Range ($533-$1067) | * Personal Communication with SSPs |
| Indirect costs | Funds used for indirect costs that might occur at the program. | $10,000 per year average, which will be the cost for medium. Small/Large will be made proportional to medium cost. Small ($2000), Medium ($10000), Large ($20000) | * Personal Communication with SSPs, ranges are estimates |
| Postage and shipping | Shipping costs of harm reduction supplies and other mailing needs including shipping of specimens, ranges are estimates. | $150-$1200 per year as average cost, which will be the amount for medium size SSP. Small/Large will be made proportional to medium cost using size of client base. | Estimation |
| Website hosting | Funds used to purchase and maintain program website | Ranging from $10-$40 per month | Google search (average cost of website hosting); estimation of values. Link: <https://lawyerist.com/67674/calculating-monthly-website-fees-are-you-getting-gouged/> Access Date: 19Jul2017 |
| Electronic Data Capture System | Used to track client data | $2700 (excluding taxes) | Quote from Neo360 |
