# Supplementary material for: Estimated cost of comprehensive syringe service program in the United States
Source: PLoS One. 2019 Apr 26;14(4):e0216205. doi: 10.1371/journal.pone.0216205 (PMC6485753; doi:10.1371/journal.pone.0216205)
Supplement: S4 Appendix — (DOCX) [file pone.0216205.s004.docx]

**S4 Appendix.** **Prevention/medical services cost methods and sources**

| **Prevention/medical Services Costs** | **Quantity and Justification** | **Estimated Unit Cost** | **Source** |
| --- | --- | --- | --- |
| Male and female condoms | Estimated 2-3 condoms per client per week. Total of 104-156 condoms per client per year. | $0.08-$0.12 per condom | Safety Works Harm Reduction Supplies website. Link: <http://www.exchangesupplies.org/> Access Date: 19Jul2017 |
| Needles/syringes | Assuming one syringe per injection use per client. 400-800 needles per client per year. Range depends on type of drug injected, injection frequency of the client, and published estimate of annual number of injections among US PWID | $0.15 per syringe | Estimation |
| Cotton filters | One cotton per injection. 400-800 cottons per client per year. Large SSP (1,000,000-2,000,000), Medium SSP (500,000-1,000,000), Small SSP (100,000-200,000) | $13.31 per 4000 cotton balls as of 02/03/2016 from First Option Medical Website. Large SSP ($3327.50-$6655.00), Medium SSP ($1663.75-$3327.50), Small SSP ($332.75-$665.50) | First Option Medical website. Link: <https://www.firstoptionmedical.com/kendall-cotton-ball-medium-rayon-nonsterile-pack/ecomm-product-detail/190728/?gclid=CPGa-sO8gdICFZqLswodY8MLHw> Access Date: 19Jul2017 |
| Sterile water | One vial per one syringe so 400-800 vials | $109 per box (1000 5-mL vials/box) | Safety Works Harm Reduction Supplies website. Link <http://www.exchangesupplies.org/> Access Date: 19Jul2017 |
| Alcohol swabs | Assuming 400-800 pads per client (one for each injection). | $96 per box (8000 pads/box) | Safety Works Harm Reduction Supplies website. Link <http://www.exchangesupplies.org/> Access Date: 19Jul2017 |
| Cookers | Two cookers per every 10 syringes (estimated). Will give out from 80-160. | $160 per box (1000 units/box) | Exchange Supplies website. Link: <http://www.exchangesupplies.org/> Access Date: 19Jul2017 |
| Twist ties | Estimated one twist tie per cooker give out; 80-160 twist ties per client per year | $8 per 2000 twist ties | Staples website. Link: <http://www.staples.com/> Access Date: 19Jul2017 |
| Tourniquets (non-latex) | Estimated 20 tourniquets per client each year | $208 per box (1000 units/box) | Safety Works Harm Reduction Supplies website. Link <http://www.exchangesupplies.org/> Access Date: 19Jul2017 |
| First aid/wound care supplies | Estimating one wound care kit for 40-60% of clients. In Study to Assess Hepatitis Risk (STAHR) unpublished data, 47% of young PWIDs ever have an abscess and 23% had an abscess in the past 3 months. 71% reported more than one abscess. | $129 per 50 wound care kits | Safety Works Harm Reduction Supplies website. Link <http://www.exchangesupplies.org/> Access Date: 19Jul2017 |
| Naloxone | Estimated giving to 80-100% of clients. Give out two units per client. In STAHR 28.1% of participants had experienced at least one episode of overdose. Also need to account for non-injectors (family and friends of injectors) who want nalaoxone | $20-$40 per unit | NPR Health. Link: <http://www.npr.org/sections/health-shots/2015/09/10/439219409/naloxone-price-soars-key-weapon-against-heroin-overdoses> Access Date: 19Jul2017 |
| Hygiene Products | Tampons/pads - assuming 50% of clients are women and need 5 day supply (4 per day) per month. Combs (small containers) - 3 per client/year. Toothpaste (small containers) - 25 per client/year. Toothbrush - 10 per client/year. Lotion (small bottles) - 20 per client/year. Shampoo (small bottles) - 20 per client/year. Soap (tiny soap bars) - 30 per client/year. | Tampons - $11.99/50 count. Pads-$18.32/240 count. Comb - $8.99/72 count. Toothpaste - $26.08/144 count. Toothbrush - $26.99/100 count. Shampoo - $19.99/72 count. Soap - $41.99/400 count. | Tampons - Amazon ("Tampon Bulk"; Pads - Amazon ("Pads Bulk"); Combs - Amazon ("Small Combs bulk”); Toothpaste - Amazon ("Travel Size Toothpaste Bulk"); Toothbrush - Amazon ("Toothbrush Bulk"); Shampoo/Soap - Amazon ("Travel Size Shampoo"). Link: <https://www.amazon.com/> Access Date: 19Jul2017 |
| Hazardous waste disposal | Funds used to remove hazardous waste from program building, including used syringes. A 30-gallon container is used to collect all of the 1-quart containers given out to clients. 30-gallon containers can take up to 40 1-quart containers. Return Shipping is included in the 30-gallon container cost. For Large SSP, 500-1000 30-gallon containers for disposal; Medium SSP will need 250-500 30-gallon containers; Small SSP will need 50-100 30-gallon containers. Estimates are based off number of clients per SSP size and how many syringes each client will use per year. | 30-gallon containers (including return shipping) will cost $249 (including $40 estimation for shipping). | Sharps Compliance Inc. website. Link: <http://www.sharpsinc.com/> Access Date: 19Jul2017 |
| Sharps disposal containers | Funds used to purchase sharps containers to be given out to clients to collect used syringes. 1-Quart sharp containers used in this calculation. Estimates around 50 syringes per 1-quart container. For Large SSPs, will need 20,000-40,000 1-quart containers; Medium SSP will need 10,000-20,000 containers; Small SSP will need 2000-4000 1-quart containers. | Cost of 1-quart containers are $290 for 120. | Amazon search.  Link: https://www.amazon.com/ Sharps-Container-Biohazard-Needle-Disposal/dp/B00XWC2GKU/ref=sr_1_3_a_it?ie=UTF8 &qid=1483635647&sr=8-3&keywords=sharps+container+1qt  Access Date: 19Jul2017 |
| Vitamin C | 1 sachet per cooker | $50 for 1000 sachets | Exchange Supplies website. Link: exchangesupplies.com Access Date: 19Jul2017 |
| Zip bags (for cottons) | Estimating 10 cottons per bag | 1000 units/ box @ $30/box | Safety Works Harm Reduction Supplies website. Link <http://www.exchangesupplies.org/> Access Date: 19Jul2017 |
| Paper bags | 1 bag per client for supplies obtained at SSP | 1000 units/ box @ $31/box | Safety Works Harm Reduction Supplies website. Link <http://www.exchangesupplies.org/> Access Date: 19Jul2017 |
