# Supplementary material for: Estimated cost of comprehensive syringe service program in the United States
Source: PLoS One. 2019 Apr 26;14(4):e0216205. doi: 10.1371/journal.pone.0216205 (PMC6485753; doi:10.1371/journal.pone.0216205)
Supplement: S5 Appendix — (DOCX) [file pone.0216205.s005.docx]

**S5 Appendix.** **Onsite medical/testing services cost methods and sources**

| **Onsite medical/testing services** | **Quantity and Justification** | **Estimated Unit Cost** | **Source** |
| --- | --- | --- | --- |
| HIV counseling and testing | Rapid HIV oral test, along with counselling (risk assessment and harm reduction recommendations); Estimate ranges from low assumption of 30% of clients being tested to high assumption of 80% being tested | $18/unit | MedEx Supply website. Link: medexsupply.com Access Date: 19Jul2017 |
| HCV counseling and testing | Rapid HCV test, along with counselling (risk assessment and harm recution recommendations); Estimate ranges from low assumption of 30% of clients being tested to high assumption of 80% being tested | $25/unit | MedEx Supply website. Link: medexsupply.com Access Date: 19Jul2017 |
| Pregnancy test | one test per female per year, for 50% of the total population. | $12/25 tests | Amazon search. Website: [https://www.amazon.com/s/ref=nb_sb_ss_c_2_14?url=search-alias%3Dhpc&field-keywords=pregnancy+test&sprefix=pregnancy +test%2Caps%2C140&crid=1R6SEY37LNFIB](https://www.amazon.com/s/ref=nb_sb_ss_c_2_14?url=search-alias%3Dhpc&field-keywords=pregnancy+test&sprefix=pregnancy%20+test%2Caps%2C140&crid=1R6SEY37LNFIB)  Access Date: 19Jul2017 |
| HBV vaccination | 100% first dose, 50% second dose, and 25% third dose | $52-$62 for 10 pack | CDC vaccine price list. Website: <http://www.cdc.gov/vaccines/programs/vfc/awardees/vaccine-management/price-list/>. Access Date: 19Jul2017 |
| HAV vaccination | 100% first dose, 75% second dose | $62-$67 for 10 pack | CDC vaccine price list. Website: <http://www.cdc.gov/vaccines/programs/vfc/awardees/vaccine-management/price-list/>. Access Date: 19Jul2017 |
