# Supplementary material for: Estimated cost of comprehensive syringe service program in the United States
Source: PLoS One. 2019 Apr 26;14(4):e0216205. doi: 10.1371/journal.pone.0216205 (PMC6485753; doi:10.1371/journal.pone.0216205)
Supplement: S6 Appendix — (DOCX) [file pone.0216205.s006.docx]

**S6 Appendix.** **Mobile van cost methods and sources**

| **Mobile Van Costs** | **Quantity and Justification** | **Estimated Unit Cost** | **Source** |
| --- | --- | --- | --- |
| Mobile van costs | Used to reach more clients away from office, increasing the coverage of the SSP. Transports supplies and staff. | $20,000-$60,000 | RV trader website. Website: (<http://www.rvtrader.com/>. Link: 19Jul2017 |
| Mobile van maintenance | Funds used for maintenance of van | $1,000-$,1250 per year | * Personal Communication with SSPs, estimation for ranges |
| Mobile van storage | Funds used to pay for storage of van | $1,000-$1,500 per year | * Personal Communication with SSPs, estimation for ranges |
| Mobile van gas | Funds used for gas; range depends on anticipate travel distance from fixed site | $,2000-$3,000 per year | Personal Communication with SSP, estimation for ranges |
| Mobile van registration fee | Funds used to pay fees for registration mobile van | $100-$200 per year | * Personal Communication with SSPs, estimation for ranges |
| Mobile van insurance | Funds used to pay insurance costs for van. Included in overall insurance above. |  | * Personal Communication with SSPs, estimation for ranges |
| Mobile Van Furniture | Used for client outreach. Includes 1 folding table, 8 folding chairs, and 1 pop-up tent. | A total of $343 per mobile van. Includes the cost of 1 folding table ($49), 8 folding chairs ($22 per table), and 1 pop-up tent ($118). | Wayfair website (is an online website and ships everything for free): <https://www.wayfair.com/>. Access Date: 19Jul2017 |
